# The Human Factor in Automated Image-Based Nutrition Apps: Analysis of Common Mistakes Using the goFOOD Lite App

**DOI:** 10.2196/24467

**Published:** 2021-01-13

**Authors:** Maria F Vasiloglou, Klazine van der Horst, Thomai Stathopoulou, Michael P Jaeggi, Giulia S Tedde, Ya Lu, Stavroula Mougiakakou

**Affiliations:** 1 ARTORG Center for Biomedical Engineering Research University of Bern Bern Switzerland; 2 Department of Health Professions Bern University of Applied Sciences Bern Switzerland

**Keywords:** mHealth, dietary assessment, smartphone, apps, human mistakes, mobile phone

## Abstract

**Background:**

Technological advancements have enabled nutrient estimation by smartphone apps such as goFOOD. This is an artificial intelligence–based smartphone system, which uses food images or video captured by the user as input and then translates these into estimates of nutrient content. The quality of the data is highly dependent on the images the user records. This can lead to a major loss of data and impaired quality. Instead of removing these data from the study, in-depth analysis is needed to explore common mistakes and to use them for further improvement of automated apps for nutrition assessment.

**Objective:**

The aim of this study is to analyze common mistakes made by participants using the goFOOD Lite app, a version of goFOOD, which was designed for food-logging, but without providing results to the users, to improve both the instructions provided and the automated functionalities of the app.

**Methods:**

The 48 study participants were given face-to-face instructions for goFOOD Lite and were asked to record 2 pictures (1 recording) before and 2 pictures (1 recording) after the daily consumption of each food or beverage, using a reference card as a fiducial marker. All pictures that were discarded for processing due to mistakes were analyzed to record the main mistakes made by users.

**Results:**

Of the 468 recordings of nonpackaged food items captured by the app, 60 (12.8%) had to be discarded due to errors in the capturing procedure. The principal problems were as follows: wrong fiducial marker or improper marker use (19 recordings), plate issues such as a noncompatible or nonvisible plate (8 recordings), a combination of various issues (17 recordings), and other reasons such as obstacles (hand) in front of the camera or matching recording pairs (16 recordings).

**Conclusions:**

No other study has focused on the principal problems in the use of automatic apps for assessing nutritional intake. This study shows that it is important to provide study participants with detailed instructions if high-quality data are to be obtained. Future developments could focus on making it easier to recognize food on various plates from its color or shape and on exploring alternatives to using fiducial markers. It is also essential for future studies to understand the training needed by the participants as well as to enhance the app’s user-friendliness and to develop automatic image checks based on participant feedback.

## Introduction

### Background

Around 12% of mobile health (mHealth) apps have a *wellness* focus on nutrition and diet. These apps often enable users to capture their food intake and receive recommendations for a healthy diet [1]. Sharp et al [2] reviewed comparative studies on methods of recording diets; they found that most participants preferred procedures that included dietary assessment methods based on a mobile phone rather than conventional reference methods [2].

These new technologies have several advantages over conventional methods. First, they do not rely on respondents’ memory, but they can provide users with automatically processed data and real-time advice [3]. Second, they use portable devices and have better social acceptance than conventional methods of dietary assessment [4]. Researchers also benefit from smartphone app-based methods, as apps can decrease workload, reduce printing and postage costs, lower the risk of transcription errors [5], and optimize space and security aspects required for paper file storage [6]. Owing to their feasibility and cost-effectiveness, innovative mobile phone–based tools may be superior to conventional tools in large-scale setups [7].

Image-based apps can be divided into 2 broad categories. The first one includes the majority of the existing apps, which are either (1) manual, in which no artificial intelligence (AI) component is integrated and the user inserts manually both the type and portion size of food or drink, or (2) semiautomatic, in which some type of AI features are integrated, for example, automatic food recognition, but the portion size estimation is manually provided by the user [8]. The second category includes systems that are fully automatic based on AI approaches [8]. Systems of the first category usually require the user to manually enter the food item, while often they use either barcode scanners to recognize packaged food labels [9] or algorithms for the automatic recognition of food items from images. Once food items have been recognized, the user is typically asked to enter portion size or volume by hand, so that the system can convert this input into nutrient information. However, tools in this category are usually not validated or certified, the number of food categories they support is limited, and it is not always clear which nutrient database is used for nutrition information [10]. Another drawback of this category is that individuals may inaccurately estimate the portion size [11]. This is a significant problem and accounts for nearly 50% of the mistakes in the food records of dietary assessment apps [12]. The systems in the second category (totally based on AI) use food image or video input [13-17] automatically and in real time to (1) identify and segment the different food items, (2) recognize each type of food item, and (3) create a 3D model of all individual food items. The conversion of food images or videos to calories or macronutrient content is supported by food composition databases (eg, United Sates Department of Agriculture [USDA] nutrient database and Swiss food composition database) [8]. The primary limitation of this category is that some food types, such as mixed foods (eg, lasagna) or beverages, are challenging to analyze [13], as they usually contain ingredients that are not clearly identifiable or that are placed in containers, which makes it difficult to assess volume.

Owing to the use and continuous improvement of mHealth and AI technologies, it is likely that they will replace paper-and-pencil methods altogether. Currently, nutritional scientists studying dietary assessment by apps mainly focus on comparing innovative with conventional methods for dietary assessment, whereas computer scientists mainly focus on optimizing the algorithms. However, to achieve high-quality data, both nutrition and computer scientists also need to focus on the behavioral aspects of data acquisition. In an international survey conducted among health care professionals (n=1001) in 6 continents, they mentioned that to recommend a *Nutrition and Diet* app to their patients or clients, they would prefer an app that is easy to use (87.1%), validated (68.1%), supports automatic food recording (56.5%), and automatically outputs nutrient estimations (52.4%) [18]. However, accurate data for food quality assessments can only be based on the correct capture of meal images, which is of vital importance if smartphones are to be used as a reliable source for food records. Thus, the quality of the captured data can severely affect the quality of the assessment, and it is indicated that correct data capture is a critical factor if the app is to be properly used and is to provide the most accurate results. Rather than removing erroneous data from studies, in-depth analysis should help explore common mistakes and thus further improve automated apps to assess nutritional intake.

### Objectives

Along these lines, the aim of this study is to explore and evaluate common user errors made when using the goFOOD Lite app for collecting dietary intake data, considering that the collected data are used for automatic food recognition and nutrient estimation (goFOOD). Thus, mistakes can potentially influence the automation of the process. The results will help improve the instructions given, adjust the app to the user needs, and enhance the overall automatic functionalities of the app.

## Methods

### Recruitment and Screening Procedure

#### Sample Inclusion and Exclusion Criteria

Eligible volunteers for the study included adults (18 years and older) from the general population with self-reported adequate literacy in information communication technology, that is, they knew how to use a smartphone, who also provided written informed consent before the start of the study. Dietitians, nutritionists, and students in the fields of nutrition and dietetics were excluded to avoid bias related to profession. No prior familiarity with the app was needed to be eligible to participate. The participants did not perform any nutrient estimation; hence, they were not required to be experienced in this subject.

#### Participants

A convenience sample was recruited following the snowball method, that is, starting with acquaintances of members of the team at Bern University of Applied Sciences and the Artificial Organ (ARTORG) Research Center of Biomedical Engineering (University of Bern). In addition, students who had been informed of our study through our communication campaign via promotional flyers at clinics, the campus of Bern University of Applied Science, and social media were enrolled.

#### goFOOD System

goFOOD is an Android system that supports both images and video as an input to automatically determine the type, volume, calories, and macronutrient content (carbohydrates, fat, and protein) of a meal by using AI and computer vision–based analysis of images acquired by the users’ smartphones [19].

The version of interest in this study is the one using 2 meal images as input, captured from different viewing angle, and a reference card that must be placed next to the meal during the image capturing. The image processing module of the system consists of the following 3 main stages: (1) food segmentation, (2) food item recognition, and (3) volume estimation. Deep neural networks are applied to process the captured food images, and this performs food segmentation and recognition [20,21], whereas a 3D reconstruction–based algorithm estimates food volume [17]. The meal’s calorie and macronutrient content are then calculated on the basis of each food category, volume, and food composition database [22,23]. goFOOD supports 319 fine-grained food categories and has been validated technically.

#### goFOOD Lite and Participants’ Actions or Walkthrough Process

For the purpose of this study, a simplified version of goFOOD, called goFOOD Lite, was developed. With goFOOD Lite, the users can record their meal (food or beverage), but—unlike in the original goFOOD app—the Lite version does not provide any estimated results (eg, nutrient content and portion size) to the users as would have been the case for collecting dietary intake on the population level. With this app, participants could record their food intake by taking 2 photographs at specific angles of their meal before and after eating. This version of the app informs the user of the correct positioning (angle) of the phone for photo and input. As in other apps, goFOOD Lite uses a specially designed reference card as a fiducial marker that must be placed next to the recorded item to ensure that the 3D volume estimation is accurate. Moreover, for the images to be valid, the following criteria must be met:

1. The recorded foods are best positioned within an elliptical plate, either neutral (white) in color or with high color contrast to the background. Though this is not an absolute requirement, the participant is urged to comply, as this facilitates subsequent processing, that is, nutrient estimation.
2. The recorded item must be fully visible in the image. If it is a plated meal, then the entire plate must be in the image. If it is a nonplated meal, then the entire items must be within the image. If it is a beverage, then the entire glass or bottle containing the beverage must be within the image.
3. A special reference card (the size of a credit card) used as a fiducial marker is provided to the participant. This card must be used for all recordings of foods or beverages, as it is of vital importance for the estimation of food volume and the subsequent nutritional analysis. The card must be placed on the same table or surface as the items being recorded and has to be fully visible in all images from its top side, with the most colors and textures. Loyalty cards of large supermarket chains can also be used instead of the designated reference card.
4. The relative position of the recorded item and the card must not change between the 2 different lateral images.
5. The foods and beverages must be recorded separately.

**Figure 1:**
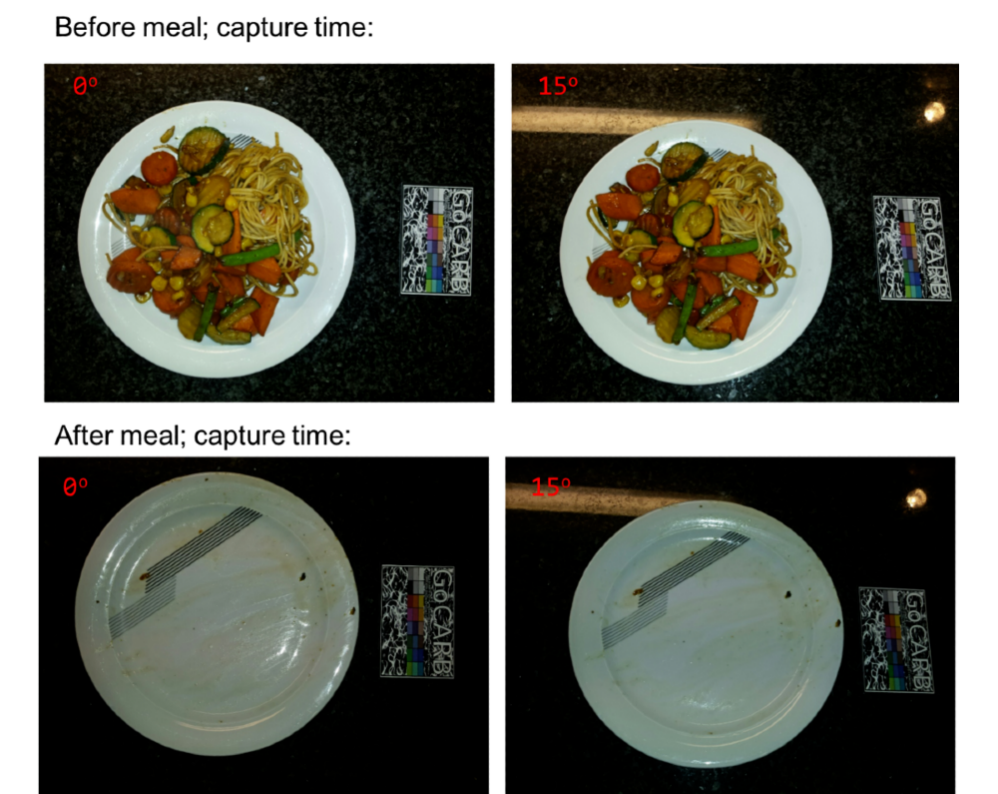
Example of 2 correct food recordings: 1 recording before and 1 recording after consumption.

Every consumed food (plated, nonplated, or packaged) and beverage needs to be recorded. More specifically, the participants were asked to record their food and beverages before and after consumption. One recording comprises 2 images of the corresponding item or items, that is, the food or beverage. These 2 images are captured from different viewing angles, as indicated and guided by the app (0° and 15° to the surface or table). The recording also contains the creation time and date. An example of 2 correct recordings is shown in Figure 1.

The specific procedure for each recording was as follows:

1. The user indicates if the recording is for a food or a beverage.
2. The user indicates if the recording occurs before or after consumption.
3. The user captures 2 images at specific angles. The app has a feature that guides the user toward the correct angle, and if this angle is not met, then it is not possible to capture the image.
4. The app attempts to transmit the recording to the server.
  - If no internet connection is available, then the user is prompted with an informative message urging them to ensure that the phone has internet access and to attempt to record again. The recording is not completed and is not transmitted or stored.
  - If a working internet connection is available, the recording is transmitted and stored on the server. The recording is completed.

If the procedure does not reach step 4b, then it is not considered complete and is not stored.

### Study Procedure

Study participants were asked to use the goFOOD Lite app to record all foods and beverages consumed during a period of 24 hours.

Participants took part in 1 of the 3 different instruction days to be informed about the aim of the study, how the goFOOD Lite app works, and to sign consent forms. The procedure and criteria presented in the previous section (*goFOOD Lite and Participants’ Actions or Walkthrough Process*) were clearly explained to the participants as well as the correct side of the reference card; this is part of the recording procedure but has not yet been specifically mentioned.

The participants were guided through the app’s functions by watching several demos and by trying the app themselves and asking questions. Those unable to be present on the designated introduction days received personal instructions from one of the scientists responsible for data collection. At the end of each instruction day, all participants were asked to sign a consent form. No compensation was provided to the participants. Every participant was provided with the following:

- An Android smartphone with a preinstalled goFOOD Lite app and a functioning 4G internet connection
- A designated reference card
- Instructions for returning the phone at the end of the 24-hour test period
- Written instructions and a demonstration video to ensure the proper usage of the app

The participants had to provide basic personal data (address, email, and telephone number) on the instruction days, as this was required for sending the smartphones and the delivery of the log-in data and the instructional video.

The list of instructions provided to the participants, as a summary, is as follows:

1. Produce a recording of every food or drink you consumed within a period of 24 hours.
2. Produce a recording before and after consuming the food or drink.
3. Make sure that the phone has internet access before attempting any recording.
4. Follow the 4 procedural steps presented to you on the instruction day and given to you with the written instructions.
5. Try to follow criterion number 1 (presented in *goFOOD Lite and Participants’ Actions or Walkthrough Process* section).
6. Make sure that criteria 2-5 are met; otherwise, the recording will not be valid.

Furthermore, an email including the participant’s log-in data was sent by our team to each participant. In addition, there was no further communication unless the participant faced technical difficulties, in which case, they were instructed to contact the team for support.

### Statistical Analysis

All the images stored on the server were evaluated and categorized into one of the following mistake categories: (1) missing recording, that is, only before or only after consumption recording; (2) packaged food mistake; (3) plate mistake; (4) fiducial marker mistake; (5) combination; and so on. The chi-square test of independence was performed to examine the relation between participants who made mistakes and their sex, age, number of days since the instruction day, and whether or not the participant pursued a technical profession. RStudio (version 1.0.153, 2009-2017 RStudio, Inc) was used for data processing. Statistical significance was set at *P*=.04. Descriptive analysis, defined as mean (SD), was performed.

## Results

### Self-Reported Basic Characteristics

The study began with 50 participants, but 2 participants dropped out due to technical difficulties. The study then included 48 participants (27 men and 21 women) with a mean age of 34.2 years (SD 11.7). All participants were Caucasian German-speaking Europeans living in Switzerland. The average BMI (kg/m²) was 22.7 (SD 2.9), with 76% (38/50) of the study subjects lying within the normal range (BMI 18.5-24.9), 16% (8/50) being overweight (BMI 25.0-29.9), 2% (1/50) being obese (BMI≥30), and 6% (3/50) being underweight (BMI<18.5). The self-reported characteristics of the study participants are shown in Table 1.

**Table 1:** Characteristics of the participants (n=48).

Characteristics		Participants, n (%)
**Age (years)**		
	18-29	18 (37.5)
	30-49	24 (50.0)
	>50	6 (12.5)
**Sex**		
	Female	21 (43.8)
	Male	27 (56.2)
**Ethnicity or race**		
	Caucasian (European)	48 (100.0)
**Spoken language**		
	German	48 (100.0)
**Profession**		
	Student	9 (18.8)
	Employed	38 (79.2)
	Retired	1 (2.1)

### Food Pictures

A total of 529 food recordings were captured by the app. Of these, 9.6% (51/529) were single recordings, that is, they contained images from only before or only after the meal. However, these 51 recordings were not discarded from the automatic analysis. The remaining 478 recordings formed 239 before or after meal pairs. Moreover, 61 of the initial 529 recordings contained packaged food and were excluded from the automatic analysis, as the system did not yet support a barcode scanner when the study was conducted.

**Figure 2:**
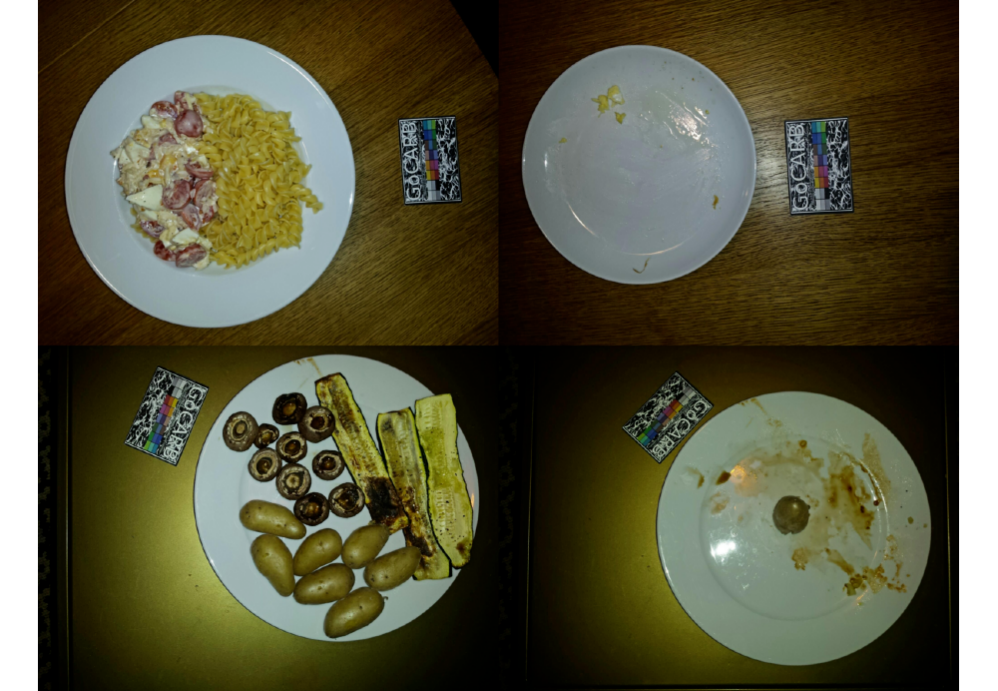
Samples of usable recordings.

Of the 468 nonpackaged food recordings, 60 (12.8%) contained mistakes and were further categorized. More details on the subcategories of the mistakes are given in the next section (*Characteristics of Errors*). In Figure 2, examples of correct and usable photos are provided. In detail, 4 recordings are shown, 2 from before the meal (left images) and 2 from after the meal (right images). Each recording is represented by 1 of the 2 (different angles) images captured. All 4 of the pictures shown in Figure 2 have a second image, captured from a different angle, but as the second angle is identical in terms of the food items and plates shown, we only provide 1 of them here.

**Figure 3:**
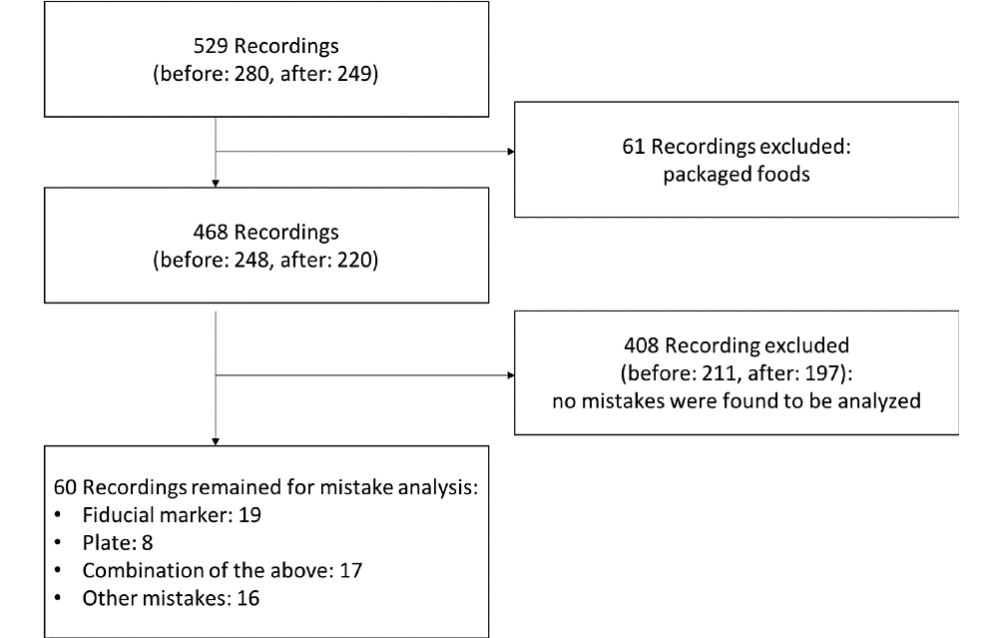
Flowchart of the data filtering process of the images obtained by goFOOD Lite (1 recording=2 photos captured at a 0° and 15° angle from the table or surface).

Figure 3 describes the process of data filtering to exclude photos that could not be processed due to errors or development stage. As mentioned in the *Methods* section, no frequent communication was planned from the members of our team with the participants, unless they faced technical difficulties.

### Characteristics of Errors

This section describes the characterization and categorization of the encountered errors.

#### Fiducial Marker Errors (n=19)

**Figure 4:**
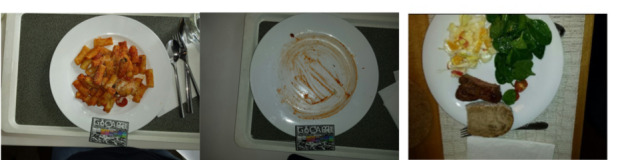
Examples of images: (a) fiducial marker (reference card) on top of the plate and (b) no fiducial marker.

To process the pictures correctly, the app requires a fiducial marker, in our case, an object the size of a credit card. In our study, the participants had to use a dedicated card created by our lab or commercial supermarket loyalty cards. The instructions given indicated that the card must (1) be placed next to the plate or food or beverage, (2) be placed on the same table or surface as the plate or food or beverage, (3) be fully visible, (4) not be moved between the 2 angle photos of 1 recording (0° and 15°), and (5) be placed with a specific side facing up (the correct side was clearly indicated). Some pictures included nonfully visible or no cards (n=9). Other issues included the use of the correct card but at the wrong side (n=2); pictures where the card was placed on top of the plate (n=2); and 6 pictures that contained mistakes outside of the aforementioned categories, such as incorrect cards or cards being moved between the 2 angle photos of 1 recording (n=6). Examples are shown in Figure 4.

#### Plate Not Fully Visible (n=8)

**Figure 5:**
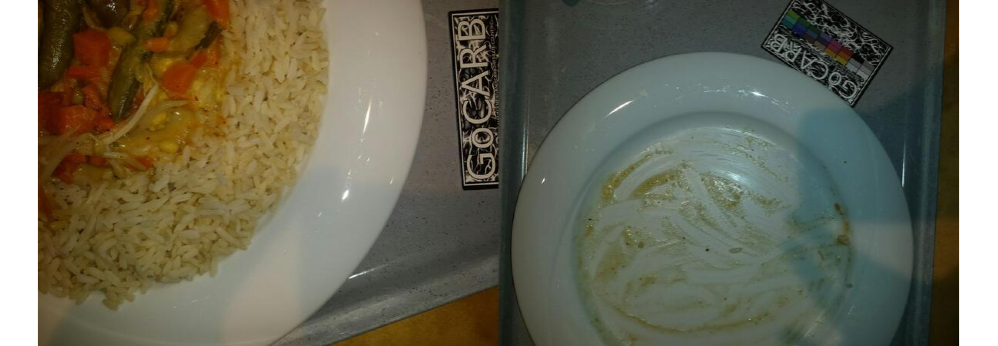
Examples of images where the plate is not fully visible.

Although participants were informed that capturing the entire plate is required for the app to function, there were nonetheless instances where the plate was not fully visible in the picture (n=8). Some examples are provided in Figure 5.

#### Combinations of Errors (n=17)

**Figure 6:**
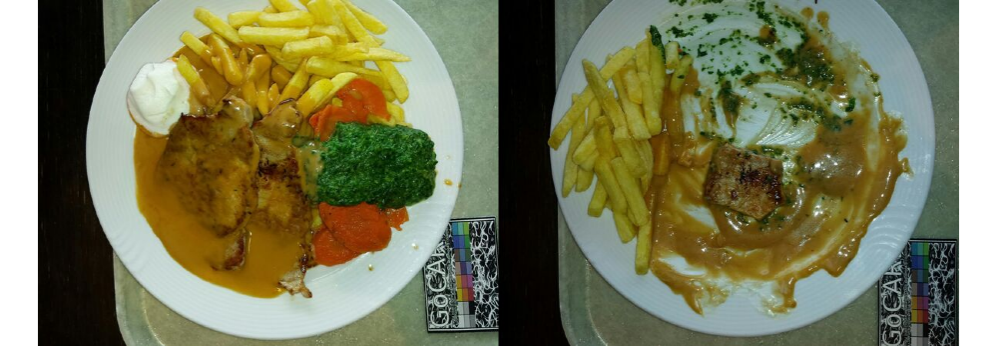
Example of combinations of errors (plate not fully visible and fiducial marker—reference card—not fully visible).

A combination of the 2 previous error categories as well as with other issues are provided in this section, and examples of which are depicted in Figure 6. Following a list of the combination of errors is provided:

- Problems with the plate plus an obstacle (n=2)
- Problems with both the plate and the card not being fully visible (n=6)
- Card not fully visible and duplicate entry of the same image (n=2)
- A picture where the card was moved between the first and second pictures and where it was not fully visible (n=1)
- A picture where the food item changes between the 2 angle pictures of the same recording and the card is placed on the plate (n=1)
- Use of an incorrect type of card combination with a plate that was not fully visible plate (n=4).

#### Other Mistakes in Photo Entries (n=16)

**Figure 7:**
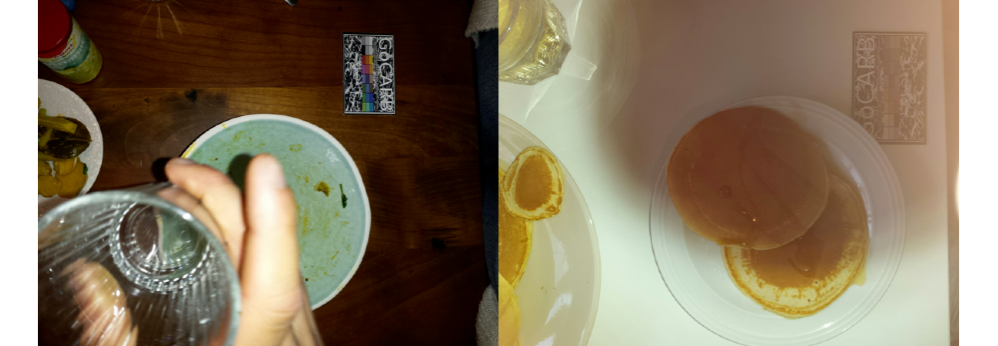
Examples of images with mistakes of (a) hand-hindering visibility and (b) shadow-hindering visibility.

Some images included an obstacle hindering the visibility of the recorded meal, such as a person’s hand (n=3). In one instance, the 2 pictures from different angles that are required for 1 recording contained different items (image at 0° was food and image at 15° was drink; n=1). Other mistakes that contained errors on files (n=2) or training testing images (n=7) were also excluded. Examples are shown in Figure 7.

#### Other Issues: Nonerrors

**Figure 8:**
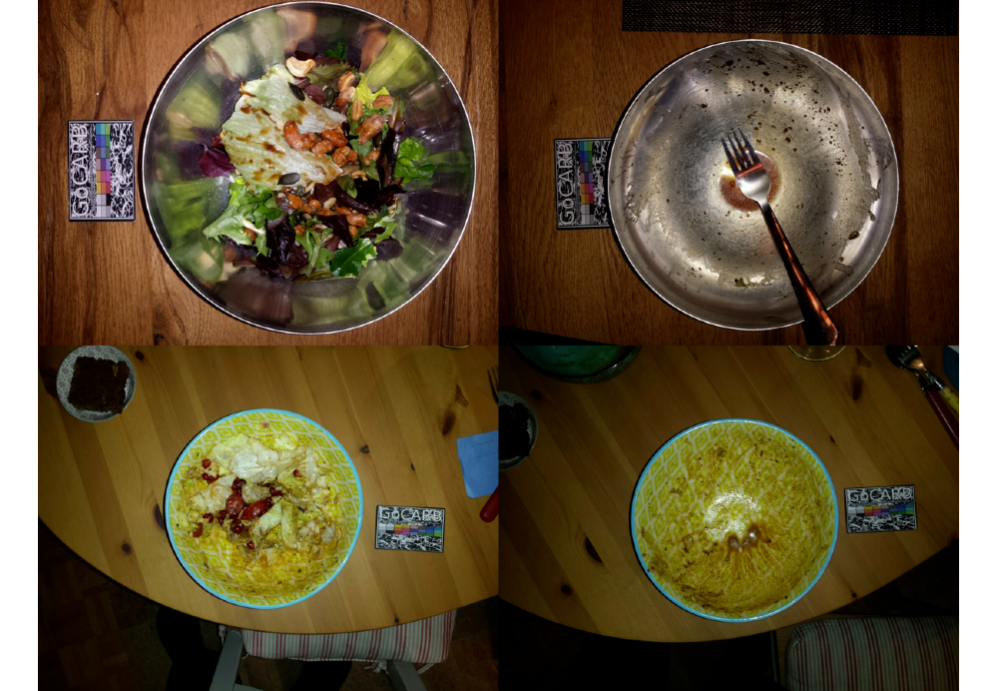
Examples of other issues: (a) wrong plate, object inside, reflections created and (b) wrong plate, highly textured.

In this section, we wish to present certain issues that cannot be considered user errors, as they have never been strictly instructed, but can affect the smooth functionality of the app. Specific issues relate to the limitations of the software or hardware. Participants were informed that the ideal scenario included elliptical white plates, but they were not strictly instructed to use plates with these characteristics. As a result, issues that posed a challenge from an algorithmic point of view included the use of plates with a nonelliptical shape, for example, rectangular, highly patterned plates, transparent plates, or those that created reflections. The highly patterned plates make it more difficult for the system to detect and recognize the food. Similarly, a plate that is not elliptical may impair overall accuracy, as in such cases, the system is required to estimate a corresponding elliptical plate. Examples of the aforementioned issues are presented in Figure 8.

**Figure 9:**
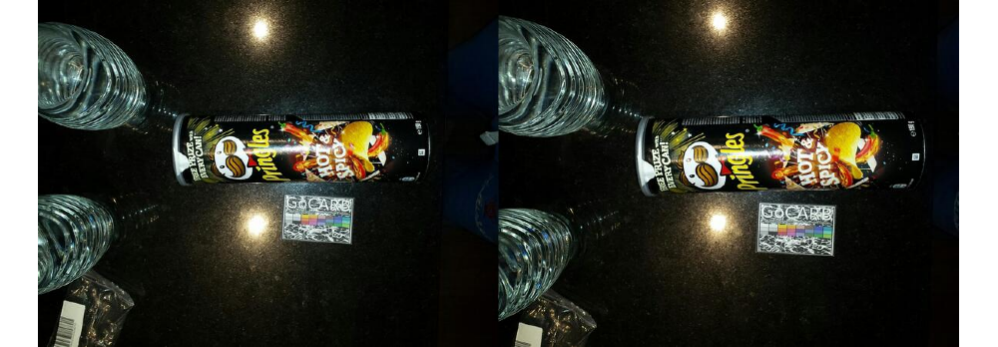
Example of mistake made while capturing packaged foods (actual food not visible).

Certain other issues are related to possible differences in the needs for instruction, as perceived by the study organizers and the end users. At the time of the study, no barcode scanner was integrated into the app. Therefore, no specific instructions were given in this respect. However, users were instructed to record all consumed items if they were part of their daily diet. By examining the recordings of packaged foods, we discovered a common problem with the images. In many cases, the before and/or after recordings contained photos showing only the packaging of the food and not its content, which is the actual food. Such an example is shown in Figure 9. This is highly problematic, as the system currently recognizes food and is not capable of recognizing brands and/or read barcodes. However, even if the system had been able to recognize the type of food from the barcode, these recordings would still be problematic, as it would be impossible to estimate the quantity. In this case, the recording of the meal both before and after consumption would be useless.

The same issue was encountered in certain beverage recordings. A number of these contain recordings of opaque bottles, where the contents, that is, the actual beverage, as well as the quantity were not visible.

### Demographic Characteristics of the Participants Making Errors

Our analysis of the errors revealed that 52% (25/48) participants made at least one error, whereas 4 participants made 4 errors and 1 participant made 6 errors. Of the participants who made errors, 13 were women and 12 were men. By age, 72% (13/18) of people aged 18-29 years made mistakes, 42% (10/24) of those aged 30-39 years made mistakes, and finally 33% (2/6) of those aged over 50 years made mistakes. However, when we checked for associations between those who made mistakes in relation to their sex (*P*=.65), age (*P*=.38), technical knowledge (*P*=.22), or days passed from the instruction day (*P*=.65), no statistically significant differences were detected.

## Discussion

### Principal Findings

This study analyzed human errors and other challenges caused by human factors when assessing food intake using the AI-based system goFOOD. Analysis of user errors should provide useful material for improving the app and with it, the quality and reliability of app-based diet recording. Most existing studies focus only on the validity or compare the performance of innovative and conventional methods, for example, apps versus manual food logs for tracking food intake and dietary assessment. In those studies, participants are generally only asked about their opinion of the user experience and/or user-friendliness of such apps; however, the reasons for any errors made in recording food intake of image-based apps are usually not investigated. In our study, 12.8% (60/468) of the captured images of nonpackaged food items would have to be discarded. Moreover, 52% (25/48) of our participants made at least one mistake in photographing their food with the app, which underlines the need for improvements in automatic methods for collecting data on dietary intake as well as in the instructions and points of emphasis provided to the user.

The main errors were primarily associated with incorrect or improper use of the fiducial marker; problems with the plates used (such as incompatible or only partially visible plates); and missing recordings, that is, discordant pairs of recordings before and after consumption. Other problems were related to testing recordings, obstruction of the camera with a hand, and a combination of errors. The results obtained from the chi-square tests indicate that errors made with the app were not associated with age, technical knowledge, sex, and time since instruction. Although our sample size is small, we could suggest that advanced technological literacy does not play a role in fully understanding how to meet the image criteria required for the app to function. Although, according to chi-square tests, we found that older people tend to make fewer mistakes, in fact, we cannot draw strong conclusions related to age, as our sample of older participants is very small. Some assumptions include that older participants were possibly more cautious after receiving instructions for using the app, as they informed us that they wanted to be conscientious in their contribution to this study. Although young people are generally more familiar with smartphones, this contradictory result may be due to overestimation of their own technological literacy or difficulty when it comes to paying attention to guidelines. Furthermore, our findings imply that immediate initiation of food and beverage consumption recording after the instruction day does not influence the number of mistakes or the correct use. As a consequence, further studies can consider the use of an image-based log app any time after the instruction days.

### Comparison With Prior Work

Other studies have been conducted where the user aspect of a nutrition app was mentioned, but without fully analyzing or providing a goal for using these observations. A study with adolescents (n=18) tested their amenability in free-living conditions with limited parental input. They were asked to use the FRapp to record their dietary intake. This app uses inputs such as images, text, voice, barcodes, and a selection of recently consumed food sets. After indicating consumed food sets, the user must insert the type and amount of food consumed. The authors suggested that only a minority of participants followed all directions [24]. In another study, the participants used the mpFR app to acquire images both before and after consumption of meals and beverages. This app uses semiautomatic food recognition, automatic volume estimation, and semiautomatic nutrient estimations [25]. An additional app study using mpFR was conducted. This included adolescents and their parents, and participants were requested to capture before and after images of their food and beverages [26]. Several findings of these studies are discussed in the following paragraphs.

### Before and After Images

In our study, 9.6% (51/529) of the images obtained from the app had only one recording from either before or after the meal. Our results are in line with other studies in which participants were asked to capture 2 separate meals (each before and after consumption) to enter in a dietary assessment app. Participants included both before and after pictures in 80% of entries for the first meal and 84% of entries for the second meal [25]. In a study with both adults and adolescents, it was found that adults were more likely than adolescents to capture images both before and after the meal and to include all the foods and beverages consumed [26]. Furthermore, in a study by Casperson et al [24], participants had difficulties in remembering to capture a postmeal image, which implies that this requirement is problematic. To help remind people to take the postconsumption image of a meal, it might be beneficial to have personalized reminders such as timing functions accompanied by sounds or vibrations or pop-up messages, as this could possibly reduce the omission of *after consumption* images.

### Fiducial Marker

Despite taking earlier user feedback into account in our study design, a total of 19 errors related to the fiducial marker still occurred. In our study, the number of recordings that did not include the entire fiducial marker was small (1.7%). However, other researchers have reported errors with fiducial markers due to participants having difficulty with the size of the card. Daugherty et al [26] asked adults (n=57) and adolescents (n=78) to record 1 or 2 meals under uncontrolled conditions, and they noted that the fiducial marker required (checkerboard square) was too large and, as a result, was sometimes partially covered by a plate or utensil (98/156). With regard to the type of fiducial marker preferred, the majority of adults (91%) and adolescents (67%) mentioned that a credit card–sized object would be easier to use and carry. The respective percentages for a USB-sized fiducial marker were 42% (30/78) for adults and 67% (38/57) for adolescents.

Another analysis from the same study with the same adolescent sample (n=70) indicated that only 23% to 29% of participants did not include the entire fiducial marker in both their before and after images [25]. According to the researchers, these mistakes arose because some participants were too short to capture the entire meal correctly. Moreover, they pointed out that even with repeated use of the app, no significant change was noted in the number of participants who included the entire fiducial marker. This mistake can be avoided or alleviated by adding a screen notification reminder on the use of the fiducial marker [22,26]. Another possible solution could be an automatic detector, which will verify in real time whether or not the marker is within the camera frame and thus inform the user. However, with the new generation of smartphones equipped with 2 or more rear cameras, the fiducial marker can be eliminated, as 2 images can be captured simultaneously [19,27].

### Other Issues

With regard to the pictures that contained only part of the plates, as well as the problems mentioned for packaged foods and beverages, we theorize that the users had misunderstood how the volume estimation works or were absolutely unaware that it even occurs. The most probable scenario is that they did not critically assess the importance of capturing the entire content, but only the packaging, or even part of it.

For the case of partially captured plates, we suppose that in our study, the users misunderstood the way the system processes the images, which requires the actual estimated food or beverage to be visible, so that its volume can be estimated. The same issue comes up quite often with the beverage recordings, where there are several recordings showing simply an opaque bottle whose content is unknown. We also believe that the requirements for the fiducial marker and that the entire plate should be in the photo might have posed a challenge to some users.

In future studies, the need to include the entire plate should be clearly stressed. In addition, it would be wise to present to the participants a more detailed structure of the system, explaining the step of volume estimation, so that the picture must contain everything the users are about to consume or have already consumed. Furthermore, it might be helpful if the app asked whether the user had given the answer, and if so, allow the user to modify it.

### Strengths

One of the main strengths of our study design was the ability to test the app in real-life situations. Furthermore, this is one of the few studies that analyzes errors made by the users rather than focusing on aspects related to diet or accuracy in nutrition calculation. Moreover, our participants came from different educational backgrounds (students, workers, etc) and represented a wide range of ages, with different levels of technological familiarity. Another advantage was that the participants were not asked to alter their eating patterns to participate. For example, we did not advise them to exclude from their diet any packaged foods, although our system was not ready to process such data (work in progress) when the fieldwork was done. In line with our goal of keeping the procedure as noninvasive as possible and capturing a *normal* day of eating, we opted not to send participants any individualized text push messages assisting them with the image entry process. In fact, we only sent one email communication halfway through the study after participants reported difficulties and compliance issues with using the app to our team of computer scientists. Another strength of our study is the low dropout rate (4%), which may have resulted from the continuous support by our team on any technical problems reported.

Furthermore, it could be considered a strength that objective data were used to capture entry errors because all data containing mistakes were excluded by the trained experts instead of only the participants’ subjective opinions. Contrary to another study [21], our participants were not encouraged to act without any limitations, and, for example, we did not let them take as many pictures as they wanted, as we saw this as a subjective opinion. Future research needs to combine both objective and subjective aspects to achieve a complete result. In the next stage, these studies could be compared in terms of acceptance rate, difficulties, and preferences.

### Limitations

A key limitation of our study design was that the participants recorded their intake for only one 24-hour period. As a result, the evaluation of the progression of entry errors—whether they increased or decreased over time and with increased use of the app—was not possible. It would have been especially interesting to see if errors decreased after the midpoint email communication addressing participants’ concerns over entry errors. Observing users over a longer period could help understand app acceptance over time, patterns of use, and changes to usage error rates. Another limitation of our study was the requirement of using a separate phone, as opposed to participants’ own phones to take the pictures. Using an additional phone could have led to a higher number of entry errors, owing to different and potentially unfamiliar user interfaces. Carrying another phone might also have caused discomfort. We also cannot omit the possibility that some foods and beverages were not captured, most probably those consumed in between meals (eg, ready-to-eat snacks such as fruits or beverages such as water). However, it is not known if meals were skipped or if participants chose not to record some snacks or simply forgot them. As stated earlier, the app is not yet ready to analyze packaged food and beverages because this feature is currently under development. However, a barcode scanner could easily be integrated into the app with the assistance of an appropriate web or mobile stored database. Manual selection of the consumed beverage and the respective portion size from a list of beverages could also be easily integrated into goFOOD in a further stage of the app’s development. Furthermore, technology savviness may have been an obstacle for older participants, and thus, their participation in our study was limited. As mentioned earlier, unfortunately, we did not have a representative sample of older participants, and thus, we cannot draw strong conclusions regarding age. Finally, another limitation of this study is that the sample consisted only of Caucasian, German-speaking people living in Switzerland, and thus, the results cannot be generalized to a wider population.

### Suggestions for Future Work

Our state-of-the-art study is novel because no other studies have focused on errors in data derived from the use of automatic apps for nutrition assessment. This study showed that adequate instructions may be needed to learn how to correctly use image-based apps and that general technology literacy may not be enough when it comes to these types of apps. Future improvements to the app could focus on improving the recognition of food on various types of plates, that is, with different colors and shapes, as well as exploring alternatives to the use of fiducial markers. Moreover, it is of vital importance for future studies that we improve our understanding of the users’ training needs as well as enhance the app’s user-friendliness and develop an automatic image check feature based on participant feedback. Furthermore, future studies with older participants are necessary to obtain concrete results on how age affects the number or severity of mistakes that are made using an image-based dietary assessment app.

This section outlines possible improvements to the app to reduce user errors. Improved navigation through the app, including some training material, could help the users to train and test themselves before the actual trial and thus eliminate basic mistakes. As an example, one participant mentioned “Maybe I took a photo of the meal too many different times or I did not take the correct picture because the app got stuck.” In this case, a system that checks and informs the user whether they have captured the picture correctly would be helpful. Another useful feature for the app might be the possibility of deleting an entry, especially in combination with a prompt from the app asking the user to try again because the “picture was not saved since the picture was not taken properly.” Text messages that also verify good lighting conditions would improve the image capturing process and usability of the app. A video tutorial at a variable pace could support those less apt or confident in using these kinds of apps. Furthermore, a section with frequently asked questions could help users troubleshoot on their own. Likewise, the integration of text messages at different stages in the data entry process could assist users and reduce errors. The suggestions offered here could be translated into different languages to ensure that users speaking different languages can fully understand the app prompts. Moreover, the app currently runs on smartphones using the Android operating system. The app could also be developed for the iOS system, and with this, the vast majority of smartphone users would be covered.

Finally, by analyzing user errors, we have learned the importance of integrating users in the design and development process of the goFOOD app. This makes sense, given that end users and health care professionals who benefit from such apps should have their needs considered throughout the entire development process with tools and techniques, such as extensive surveys and periodic trial tests to facilitate this process. Thus, research is essential to outline how and when these apps may most efficiently aid those needs [18].

### Conclusions

To the best of our knowledge, this is the first research study that objectively analyzes user errors in the automation of food and nutritional recognition apps in real-life conditions. Error analysis thus yields novel results, identifying many forms of human errors in different steps in the process of entering meal information into the app. The analysis of the mistakes and omissions from this study is fundamental, as the knowledge obtained can be used to optimize the different aspects of the app and to accelerate the procedure for entering meals and shed light on areas of the app and user experience that require improvements. goFOOD was designed to be a functional app that can help the process of nutritional assessment by assisting health care professionals in their everyday practice. More specifically, our hope is that nutrition apps such as goFOOD could work both as a food log (goFOOD Lite) and as a dietary assessment app, thus reducing the time and effort required by conventional methods for assessing nutrition. Moreover, the exchange of data between the user and the dietitian will facilitate their coordination in tracking food intake. Last but not least, researchers who work in the field of acquisition of nutrition data or who plan epidemiological and clinical studies will benefit from our analysis, as they can learn which data with specific characteristics (human errors) should be omitted as well as improve their understanding of mitigation controls that they can integrate to improve study planning and data quality.

## Abbreviations

AI: artificial intelligence
mHealth: mobile Health
